# Glutor, a Glucose Transporter Inhibitor, Exerts Antineoplastic Action on Tumor Cells of Thymic Origin: Implication of Modulated Metabolism, Survival, Oxidative Stress, Mitochondrial Membrane Potential, pH Homeostasis, and Chemosensitivity

**DOI:** 10.3389/fonc.2022.925666

**Published:** 2022-06-30

**Authors:** Mithlesh Kumar Temre, Saveg Yadav, Yugal Goel, Shrish Kumar Pandey, Ajay Kumar, Sukh Mahendra Singh

**Affiliations:** ^1^ School of Biotechnology, Institute of Science, Banaras Hindu University, Varanasi, India; ^2^ Deparment of Zoology, Institute of Science, Banaras Hindu University, Varanasi, India

**Keywords:** glutor, GLUT, thymoma, metabolism, cell survival regulation, targeted therapeutics

## Abstract

Neoplastic cells overexpress glucose transporters (GLUT), particularly GLUT1 and GLUT3, to support altered metabolism. Hence, novel strategies are being explored to effectively inhibit GLUTs for a daunting interference of glucose uptake. Glutor, a piperazine-2-one derivative, is a newly reported pan-GLUT inhibitor with a promising antineoplastic potential. However, several aspects of the underlying mechanisms remain obscure. To understand this better, tumor cells of thymic origin designated as Dalton’s lymphoma (DL) were treated with glutor and analyzed for survival and metabolism regulatory molecular events. Treatment of tumor cells with glutor caused a decrease in cell survival with augmented induction of apoptosis. It also caused a decrease in glucose uptake associated with altered expression of GLUT1 and GLUT3. HIF-1α, HK-2, LDH-A, and MCT1 also decreased with diminished lactate production and deregulated pH homeostasis. Moreover, glutor treatment modulated the expression of cell survival regulatory molecules p53, Hsp70, IL-2 receptor CD25, and C-myc along with mitochondrial membrane depolarization, increased intracellular ROS expression, and altered Bcl-2/BAX ratio. Glutor also enhanced the chemosensitivity of tumor cells to cisplatin, accompanied by decreased MDR1 expression. Adding fructose to the culture medium containing glutor reversed the latter’s inhibitory action on tumor cell survival. These results demonstrate that in addition to inhibited glucose uptake, modulated tumor growth regulatory molecular pathways are also implicated in the manifestation of the antineoplastic action of glutor. Thus, the novel findings of this study will have a long-lasting clinical significance in evaluating and optimizing the use of glutor in anticancer therapeutic strategies.

## Introduction

Neoplastic cells display dependence on glycolysis for their massive bioenergetic and biosynthetic requirements (1–3). Consequently, cancer cells display upregulated expression of various high-affinity glucose transporters (GLUT) to facilitate glucose uptake for accelerated glycolysis. Neoplastic cells overexpress GLUT1, GLUT3, GLUT4, and GLUT12, which vary depending on their etiology (4, 5). As glucose transporters are one of the crucial rate-limiting checkpoints of the reprogrammed carbohydrate metabolism of neoplastic cells, targeting GLUTs, particularly GLUT1 and GLUT3, has emerged as a promising antineoplastic approach (6). Thus, several inhibiting endeavors are being envisaged to imperil glucose uptake in neoplastic cells (7, 8). One of such strategies comprises GLUT inhibitors, being extensively explored for their antineoplastic potential (9). Natural products like genistein (10), quercetin (11), caffeine (12), phloretin (13), resveratrol (14), curcumin (15), and small synthetic inhibitors like cytochalasin B (16), fasentin (17), WZB117 (18), thiazolidinedione (19), STF31 (20), BAY876 (21), DRB18 (22), silybin (23, 24), and naringenin (25, 26) have been explored for their GLUT-inhibiting potential. They are also in various stages of preclinical and clinical trials (27). However, most of these inhibitors are isoform-specific and manifest antineoplastic action at higher concentrations, which can be potentially harmful to GLUT-expressing normal cells. Therefore, there is a necessity for developing GLUT inhibitors, which are expected to possess the ability for Pan-GLUT inhibition and manifest antineoplastic action at lower concentrations.

Recent reports have introduced a pan-GLUT inhibitory molecule named glutor, a piperazine-2-one derivative (**Supplementary Figure 1**), with a broad spectrum of anticancer potential, which is capable of exerting the antineoplastic action at nanomolar concentrations and is stable in an aqueous environment (22, 28–31). Moreover, glutor can synergistically interfere with tumor metabolism with glutaminase inhibitor CB-839 (29). Several aspects of the molecular mechanisms underlying its antimetabolic action remain unclear, which need to be deciphered for optimal utilization. To the best of our knowledge, no study has been conducted in this direction. It is well established that glucose metabolism can influence the chemosensitivity of neoplastic cells. However, it remains unclear if glutor can alter the tumoricidal action of chemotherapeutic agents. Furthermore, there is no report concerning the antineoplastic activity of glutor on neoplastic cells of thymic origin, which, though fatal, are rare in occurrence (32), and display elevated GLUT expression (33).

Considering the observations mentioned above, we used cells of a thymoma-derived tumor, designated as Dalton’s lymphoma (DL), which is a spontaneously originated tumor of thymic origin (34) that has been widely used as an *in vivo* tumor model for testing the therapeutic efficacy of anticancer agents (35, 36) and host–tumor relationship (35, 37). The present study reports that glutor displays a potent antineoplastic action against DL cells *in vitro* by modulating glucose metabolism, pH homeostasis, cell survival, and metabolic machinery.

## Materials and Methods

### Tumor, Mice, and Reagents

DL was used as a tumor model for understanding the effect of glutor. DL was discovered by Dr. A.J. Dalton (NCI, Bethesda, USA) as a thymoma of DBA mice with spontaneous origin (34). The DL cells were procured from the Department of Zoology, Banaras Hindu University. The DL’s ascitic growth was established by Goldie and Felix (38). Mice handling and experimental procedures were carried out according to the Institutional Animal Ethical Committee (Approval No. BHU/DoZ/IAEC/2021-2022/016). All reagents, unless mentioned otherwise, were purchased from Sigma-Aldrich, USA. Fetal calf serum was purchased from the Hyclone USA and Annexin V/PI apoptosis detection kit from Invitrogen, USA. RPMI-1640 medium (Cat. No. 31800-022) was purchased from Thermofisher Scientific, with a final glucose concentration of 11.11 mM. Primary antibodies against GLUT1 (E-AB-31556), GLUT3 (E-AB-31557), HK-2 (E-AB-14706), and LDH-A (E-AB-19937) were purchased from Elabscience USA; HIF1-α (SC-31515), C-myc (SC-40), p53 (SC-126), β-actin (SC-47778), BAX(CST 2772S), Hsp70 (CST 4872S), and MDR1/ABCB1 (CST 13978) were purchased from Cell Signaling Technology (CST) USA; VEGF (IMG-80214), TGF-β (IMG-6667-E-100), Bcl-2 (IMG-3181), and MCT1 (IMG-4021) were purchased from Imagenex, USA; PE-CD25 (55386) was purchased from BD Biosciences, USA. Secondary antibodies anti-rabbit IgG (A9919) and anti-mouse IgG (A3562) were purchased from Sigma-Aldrich, USA. RT-PCR primers were purchased from Ambion International AG, Germany, Integrated DNA Technologies, USA, and Eurofins, USA.

### Trypan Blue Dye Exclusion Test to Determine Cell Viability

The number of viable DL cells was enumerated by Trypan blue dye exclusion test following a method described earlier (35). The cell suspension was mixed with 0.4% (w/v) Trypan blue in PBS in equal volumes, and the viable cells (unstained cells) were enumerated using a hemocytometer under a light microscope (Leitz Wetzlar, Germany).

### Examination of Apoptotic Cell Population

Wright–Giemsa and Annexin V/PI staining was used to identify the apoptotic cell population. Annexin V/PI staining was carried out following the manufacturer’s instructions (Imagenex USA). Control and glutor-treated DL cells (1 × 10^6^) were washed and incubated in a working solution of 1× annexin binding buffer, followed by the addition of 10 µl of annexin conjugate and 1 µl of PI working solution (100 µg/ml) and incubation for 15 min at room temperature in the dark. The cells were then washed by centrifugation with 1× annexin binding buffer. The stained cells were mounted on a slide and observed under a fluorescence microscope. Live cells were determined to be those with weak green fluorescence, whereas the deep green high fluorescence cells were marked as apoptotic cells. Apoptotic cells were also confirmed by simultaneous examination of apoptotic morphology under phase contrast optics. To examine apoptotic cells by Wright–Giemsa staining, the cell suspension was smeared on a slide and air-dried. The cells were then fixed in methanol and stained with Wright–Giemsa staining solution. After mounting in glycerine, apoptotic cells were examined under a light microscope (Leitz Wetzlar, Germany). The apoptotic cells were identified based on the typical morphological features, including contracted cell bodies; densely stained, condensed, and uniformly circumscribed chromatin; and the presence of one or more membrane-bound apoptotic bodies containing nuclear fragments. The percentage of apoptotic cells was determined by counting more than 100 cells in at least three separate visions.

### MTT Assay for Estimation of Cell Survival

The MTT assay was performed to assess cell survival as an indicator of cellular metabolic activity, proliferation, and cytotoxicity following the method described by Mosmann (39). MTT was dissolved in PBS, and 50 µl of this solution (final concentration, 0.5 mg/ml) was added to the tissue culture plate wells containing the final concentration of 0.5 mg/ml. Cultures were incubated for 4 h at 37°C in a CO_2_ incubator to allow the formation of formazan crystals. The formazan crystals were solubilized using DMSO, and absorbance was measured at 540 nm using an ELISA plate reader (Labsystems, Finland).

### Reverse Transcriptase-Polymerase Chain Reaction

The expression of GLUT1 and GLUT3 genes were examined using a previously described method (35). cDNA was prepared using a cell to cDNA kit (Ambion, USA). The primers’ description is mentioned in **Table 1**. Thirty-five cycles of amplification were performed. Each cycle consisted of denaturation (2 min at 94°C), annealing (55–60°C) as per the genes’ primers, and elongation (30 s at 72°C). The DNA was electrophoresed on an agarose gel (2%) containing ethidium bromide (0.25% w/v) and was visualized on a UV-transilluminator. The band intensity of each gene was analyzed by ImageJ software.

**Table 1:** Primer sequences for RT-PCR analysis.

Genes	Primer sequences
GLUT1	F: 5’-CTTTGTGGCCTTCTTTGAAG-3
	R: 5’-CCACACAGTTGCTCCACAT-3’
GLUT3	F: 5’-AACAGAAAGGAGGAAGACCA-3’
	R: 5’-CGCAGCCGAGGGGAAGAACA-3’
β-Actin	F: 5’-GGCACAGTGTGGGTGAC-3’
	R: 5’-CTGGCACCACACCTTCTAC-3’

### Western Blotting

Western blotting was carried out to detect the indicated proteins following the method described by Fido et al. (40) and Goel et al. (41) with slight modifications. Cells were lysed in lysis buffer [Tris-Cl 20 mM (pH 8.0), NaCl (137 mM), glycerol 10% (v/v), Triton X-100 1% (v/v), EDTA 2.0 mM, PMSF 1.0 mM, Leupeptin 20 mM, and aprotinin 0.15 U/ml] for 30 min on ice. Protein content was measured using the Bradford method (42). Samples for electrophoresis were mixed in loading buffer [Tris-Cl 0.5 M (pH 6.8), β-mercaptoethanol 100 mM, SDS 20% (w/v), bromophenol blue 0.1% (v/v), and glycerol 10% (v/v)] and were heated for 3 min in a water bath. A sample containing 30 µg of protein was subjected to electrophoresis by SDS-PAGE. Proteins were transferred to the nitrocellulose membrane (Sartorius, Germany) at 60 mA for 1 h. The membrane was then incubated with primary antibodies against the respective proteins and secondary antibodies conjugated to alkaline phosphatase. Bands were visualized using BCIP/NBT. The band density was examined by ImageJ software.

### Estimation of Intracellular Reactive Oxygen Species

A method described by Furuta et al. (43) was used to estimate ROS expression with slight modifications. Cells (1 × 10^5^ cells/ml) were incubated with HBSS containing 0.1 mM dichlorodihydrofluorescein diacetate (DCFDA) at 37°C for 45 min. After washing with PBS, the stained cells were visualized under fluorescence optics (Nikon, Japan). The fluorescence intensity was analyzed by ImageJ software.

### Quantification of Glucose

A commercial kit from Agappe Diagnostics Ltd. (Kerala, India) based on converting glucose to H_2_O_2_ by the action of glucose oxidase was used to measure glucose content in the culture supernatant. The generated H_2_O_2_ was estimated by converting it into a red quinine product by peroxidase action. Ten milliliters of the culture supernatant was added to 1 ml of working reagent [sodium phosphate buffer (pH 7.4), phenol, glucose-oxidase, peroxidase, and 4-amino antipyrine], followed by mixing and incubation at 37°C for 10 min. Absorbance was measured at 505 nm, and the glucose content was expressed in mM.

### Estimation of Lactate

Lactate concentration in the culture supernatant was measured using an enzymatic colorimetric kit (Spinreact, Granada, Spain) based on a method described by Somoza et al. (44). Briefly, 1 ml of sample was diluted in 200 ml of PIPES (50 mM; pH 7.5) containing 4-chlorophenol (4 mM), lactate oxidase (800 U/L), peroxidase (2,000 U/L), and 4-aminophenazone (0.4 mM), followed by incubation for 10 min at room temperature, and measurement of absorbance at 505 nm. Lactate concentration was expressed in mg/dl.

### Immunofluorescent Staining of Cell Surface Molecules

Cell surface expression of GLUT1, GLUT3, and CD25 was carried out using immunofluorescence staining following a previously described method (45). After washing with PBS, the stained cells were fixed in a mixture of acetic acid and ethanol (5:95) for 10 min at −10°C. Fluorescence was visualized on a fluorescence microscope (Nikon Instruments Inc.).

### Estimation of Mitochondrial Membrane Potential by TMRE Staining

Mitochondrial membrane potential was determined following a method described by Crowley et al. by tetramethylrhodamine ethyl ester (TMRE) perchlorate staining (46). Control and glutor-treated DL cells were incubated for 20 min in a medium containing TMRE (100 nM) at 37°C. Fluorescence was detected using a fluorescence microscope (Nikon Instruments Inc.). Data are presented as the percent of TMRE high cells.

### Statistical Analysis

All experiments were conducted in triplicates. The Student’s *t*-test analyzed the statistical significance of differences between test groups. The difference was considered significant when the *p*-value was less than 0.05.

## Results

### Effect of Glutor Treatment on the Survival of DL Cells

**Figure 1:**
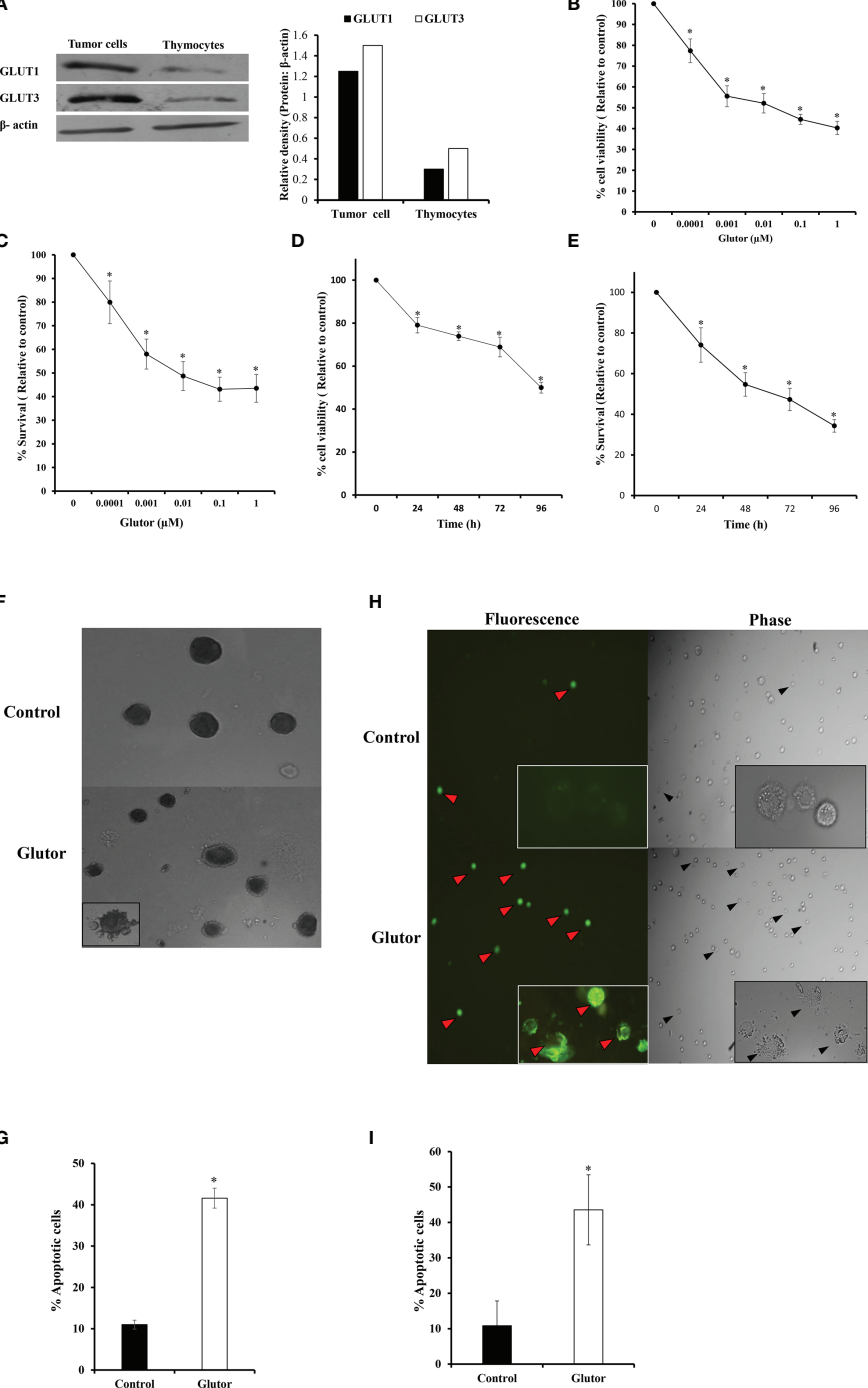
Glutor exerts cytotoxic action on DL cells. DL cells or thymocytes (1 × 10^5^) harvested from the thymi of healthy mice without tumor transplantation were processed for immunoblotting for GLUT1 and GLUT3 as described in the *Materials and Methods* section **(A)**. DL cells (1 × 10^5^) were incubated in a medium alone or containing the indicated concentrations of glutor for 24 h **(B, C)** or with 0.01 µM of glutor for the various indicated time durations **(D, E)**, followed by estimation of cell viability by Trypan blue dye exclusion assay and cell survival by MTT assay as described in the *Materials and Methods* section. DL cells incubated in a medium alone or containing glutor (0.01 µM) for 24 h were examined for the mode of cell death by Annexin V/PI **(H, I)** and Wright–Giemsa **(F, G)** staining. Values shown in **(B–E)** and **(G, I)** are mean ± SD. The plates shown in **(A)**, **(F)**, and **(H)** are from a representative experiment out of at least two experiments with similar results. The bar diagram accompanying **(A)** is the densitometry of respective bands. *p < 0.05 vs. values of the respective control.

DL cells (1 × 10^5^) and thymocytes obtained from healthy mice were examined for expression of GLUT1 and GLUT3 by Western blotting as described in the *Materials and Methods* section. Results are shown in **Figure 1A**. DL cells displayed an upregulated expression of GLUT1 and GLUT3 compared to thymocytes. Therefore, in the subsequent experiments, the effect of glutor on the survival of DL cells was explored. DL cells (1 × 10^5^) were incubated for 24 h in a medium alone or containing the indicated concentrations of glutor, followed by evaluation of cell viability by Trypan blue dye exclusion test (**Figure 1B**) and survival by MTT assay (**Figure 1C**). In another set of experiments, the DL cells (1 × 10^5^) were incubated in a medium alone or containing glutor (0.01 µM) for the indicated time durations, followed by an evaluation of cell viability (**Figure 1D**) and survival (**Figure 1E**). As shown in the results (**Figures 1B–E**), exposure of DL cells to glutor *in vitro* caused a significant decrease in viable cell count and survival in a dose- and time-dependent manner compared to untreated control. The IC_50_ was determined to be 0.01 µM. Hence, in all subsequent experiments, DL cells were treated with 0.01 µM dose of glutor for 24 h, unless mentioned otherwise, a period determined to display optimal cytotoxic action of glutor for deciphering the molecular mechanisms underlying the antitumor action of glutor. Thymocytes (1 × 10^5^) obtained from healthy mice without tumor transplantation were also incubated with various concentrations of glutor to estimate its effect on cell survival. Results indicated that glutor did not exert any cytotoxic action on thymocytes (data not shown). Given these observations that glutor can exert a tumor cell-specific cytotoxic action, the mode of observed cytostatic action of glutor was determined in the next set of experiments. DL cells (1 × 10^5^) were incubated in a medium with or without glutor (0.01 µM) for 24 h, followed by an examination of the mode of cell death by Wright–Giemsa (**Figures 1F, G**) and Annexin V/PI (**Figures 1H, I**) staining. The cells displaying features of apoptotic morphology were enumerated. The results revealed that treatment of DL cells with glutor significantly increased the number of cells exhibiting typical characteristics of apoptotic morphology compared to untreated control.

### Glutor Alters GLUT Expression in DL Cells, Accompanied by Decreased Glucose Uptake

**Figure 2:**
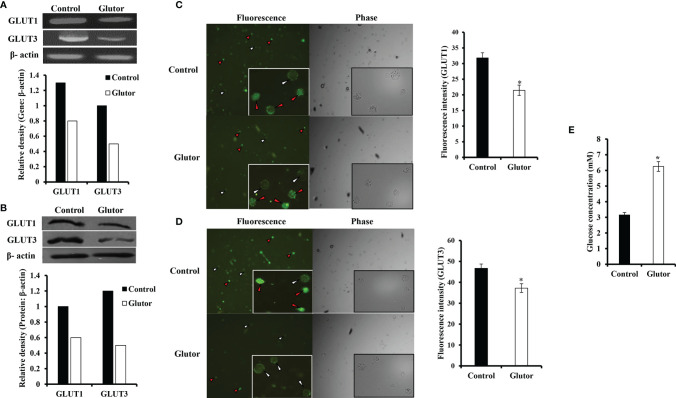
Effect of glutor on the expression of GLUT1, GLUT3, and glucose uptake by DL cells. DL cells (1 × 10^5^) were incubated in a medium alone or containing glutor (0.01 µM) for 24 h, followed by evaluation of GLUT1 and GLUT3 expression by RT-PCR **(A)** and Western blotting **(B)** as described in the *Materials and Methods* section. The expression of GLUT 1 and GLUT 3 was also examined by immunofluorescence **(C, D)**. The glucose level in the culture supernatant of control and glutor-treated DL cells was measured **(E)** as described in the *Materials and Methods* section. Values shown in **(E)** are mean ± SD. The plates shown in **(A–D)** are from a representative experiment out of at least two experiments with similar results. Accompanying bar diagrams are densitometric images of the respective bands. Bar diagrams accompanying the fluorescence images depict fluorescence intensity. *p < 0.05 vs. respective control.

**Figure 3:**
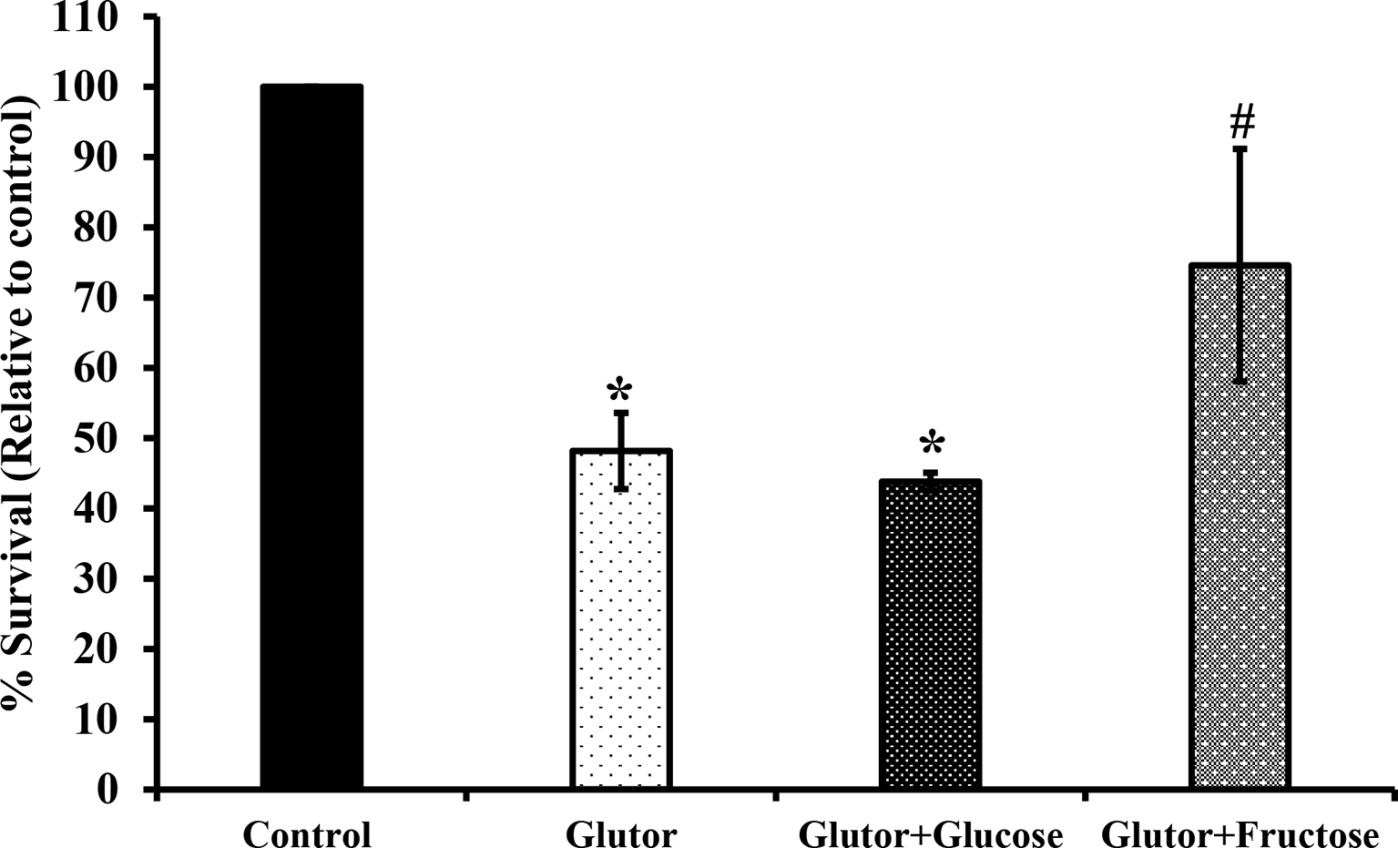
Fructose antagonizes the inhibitory action of glutor on tumor cell survival .DL cells (1 × 10^5^) were incubated in a medium alone or containing glutor in the presence or absence of glucose (1 mM) or fructose (1 mM) for 24 h, followed by an estimation of survival by MTT assay as described in the *Materials and Methods* section. Values are mean ± SD. *p < 0.05 vs. DL cells incubated in a medium alone. ^#^ *p* < 0.05 vs. DL cells incubated in a medium containing glutor or glutor plus glucose.

To understand if the tumoricidal action of glutor was accompanied by alterations in GLUT expression and glucose uptake, DL cells (1 × 10^5^) were incubated in a medium alone or containing glutor (0.01 µM) for 24 h followed by estimation of glucose level in culture supernatant and examination of GLUT1 and GLUT3 expression by RT-PCR, Western blot, and immunofluorescence microscopy as described in the *Materials and Methods* section. Results are shown in **Figure 2**. DL cells incubated with glutor displayed a decrease in the expression of GLUT1 and GLUT3 at the mRNA (**Figure 2A**) and protein levels (**Figures 2B–D**) compared to the untreated control. The glucose uptake of DL cells showed a significant decrease in the glutor-treated DL cells compared to the untreated control (**Figure 2E**). The culture medium containing glutor was supplemented with indicated amounts of glucose or fructose to understand if the anti-survival action of glutor could be reversed. Results are shown in **Figure 3**. The addition of glucose to the culture medium did not rescue the inhibition of DL cell survival by glutor, which was partially reversed by adding fructose.

### Glutor-Induced Tumoricidal Action Is Associated With Modulated Expression of Cell Survival and Metabolism Regulatory Molecules

**Figure 4:**
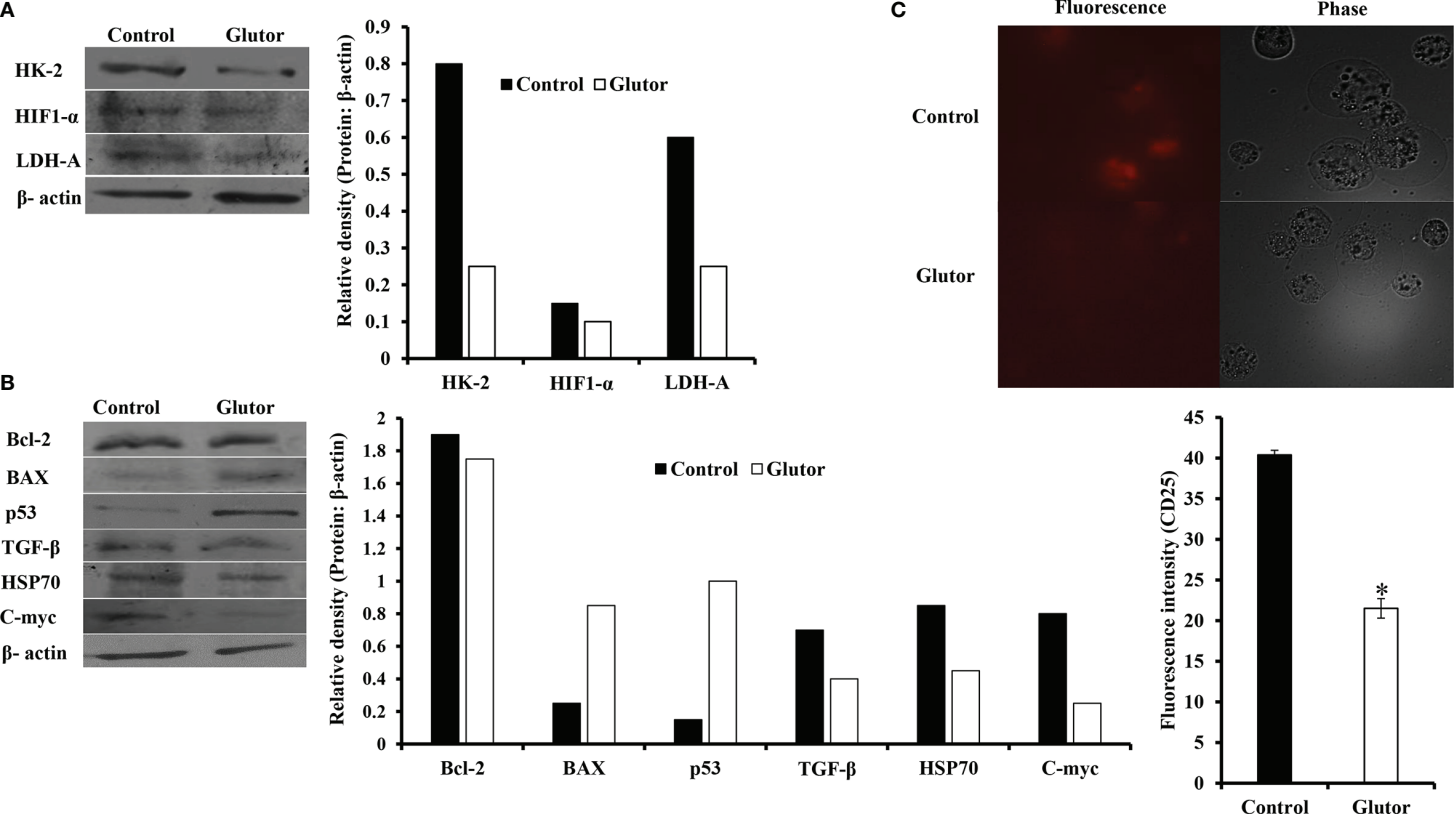
Glutor modulates expression of metabolism and cell survival regulatory molecules in DL cells. DL cells (1 × 10^5^) were incubated in a medium alone or containing glutor (0.01 µM) for 24 h, followed by Western blotting to study the expression of the indicated metabolism **(A)**, cell survival **(B)** regulatory molecules and CD25 **(C)** as described in the *Materials and Methods* section. The plates shown in **(A–C)** are from a representative experiment out of at least two experiments with similar results. The accompanying bar diagrams are densitometry of the respective bands.

In the next set of experiments, DL cells (1 × 10^5^) were incubated for 24 h in a medium alone or containing glutor (0.01 µM), followed by an examination of the expression pattern of indicated metabolism (**Figure 4A**) and cell survival regulatory molecules (**Figure 4B**) to understand the molecular mechanism(s) of the antineoplastic action of glutor. Treatment of DL cells with glutor inhibited the expression of hypoxia-inducible factor 1-α (HIF-1α), hexokinase 2 (HK-2), and lactate dehydrogenase A (LDH-A) compared to untreated control (**Figure 4A**). Furthermore, glutor treatment of DL cells also modulated the expression of cell survival regulatory molecules B-cell lymphoma 2 (Bcl-2), Bcl-2-associated X (BAX), and p53 (**Figure 4B**). The expression of Bcl-2, transforming growth factor-β (TGF-β), C-myc, and heat shock protein 70 (Hsp70) was decreased in DL cells treated with glutor, whereas the expression of BAX and p53 increased compared to the untreated control (**Figure 4B**). An immunofluorescence examination was carried out to investigate the effect of glutor on the expression of CD25. Glutor treatment of DL cells inhibited the expression of CD25 compared to the control (**Figure 4C**).

### Glutor Treatment Alters the Expression of Intracellular Reactive Oxygen Species in DL Cells

**Figure 5:**
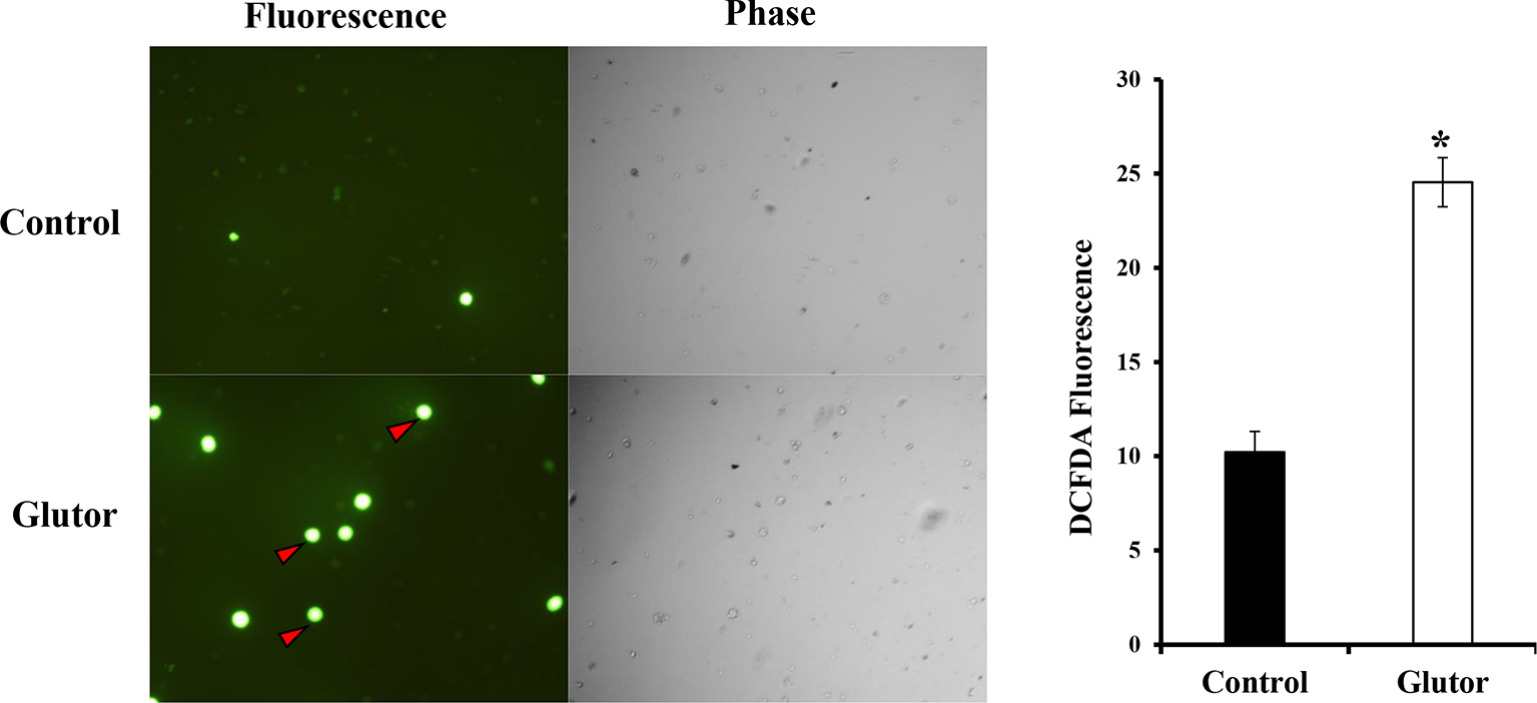
Glutor treatment elevates intracellular ROS expression in DL cells. DL cells (1 × 10^5^) were incubated for 2 h in a medium with or without glutor (0.01 µM), followed by staining with DCFDA and examination of the cells in fluorescence optics as described in the *Materials and Methods* section. The plates shown are from a representative experiment out of at least two experiments with similar results. The accompanying bar diagram depicts the fluorescence intensity of control and glutor-treated cells. Values are mean ± SD. *p < 0.05 vs. control.

Next, we checked the expression of intracellular ROS in glutor-treated DL cells. DL cells (1 × 10^5^) were incubated in a medium alone or containing glutor (0.01 µM) for 2 h, followed by an estimation of ROS expression by dichlorodihydrofluorescein diacetate (DCFDA) staining as described in the *Materials and Methods* section. Results are shown in **Figure 5**. Glutor treatment of DL cells resulted in a significantly higher ROS expression than the untreated control.

### Effect of Glutor on Mitochondrial Membrane Depolarization

**Figure 6:**
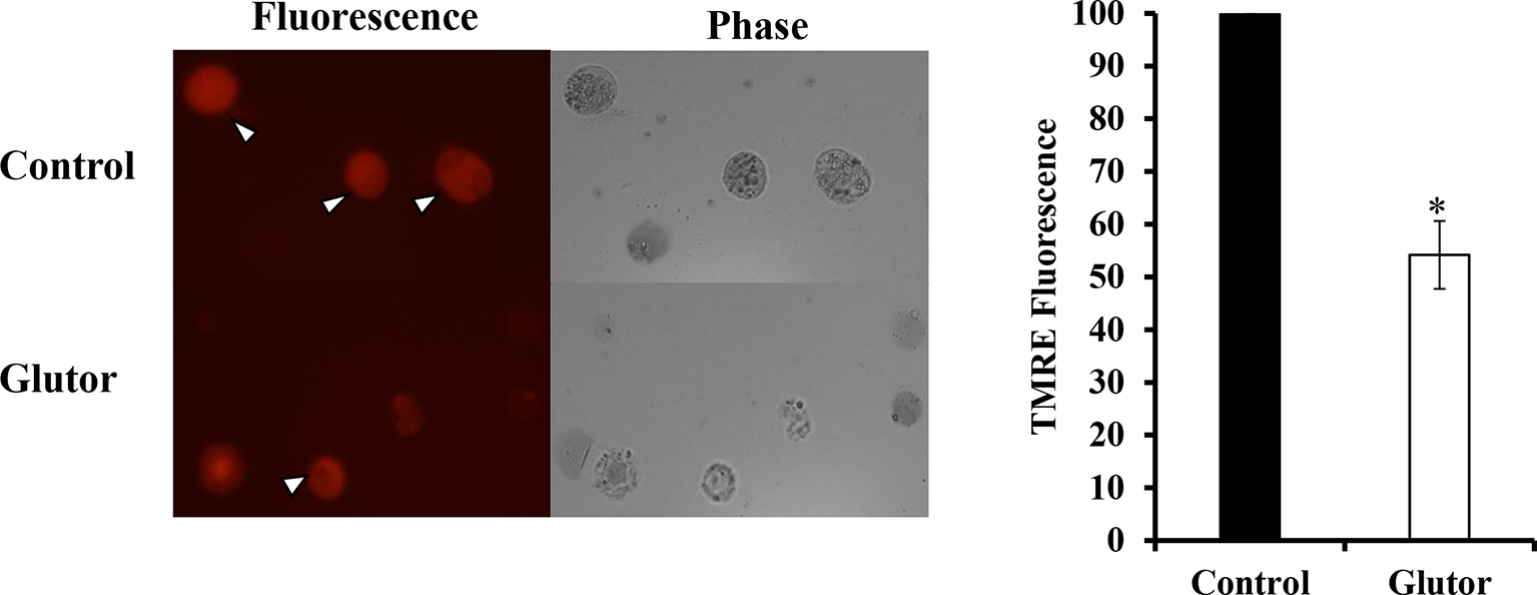
Glutor alters mitochondrial membrane potential. DL cells (1 × 10^5^) were incubated in a medium alone or containing glutor (0.01 µM) for 24 h, followed by TMRE staining as described in the *Materials and Methods* section. The plates shown are from a representative experiment out of at least two experiments with similar results. Values shown in the accompanying diagram are the mean ± SD of the fluorescence intensity. *p < 0.05 vs. control.

We also examined the effect of glutor treatment of DL cells on mitochondrial membrane potential. DL cells (1 × 10^5^) were incubated for 24 h in a medium alone or containing glutor (0.01 µM), followed by TMRE staining and observation of the stained cells under fluorescence optics. As shown in **Figure 6**, glutor treatment was found to trigger depolarization of the mitochondrial membrane as observed by inhibited fluorescence of TMRE compared to untreated control.

### Glutor Interferes With pH Homeostasis of DL Cells

**Figure 7:**
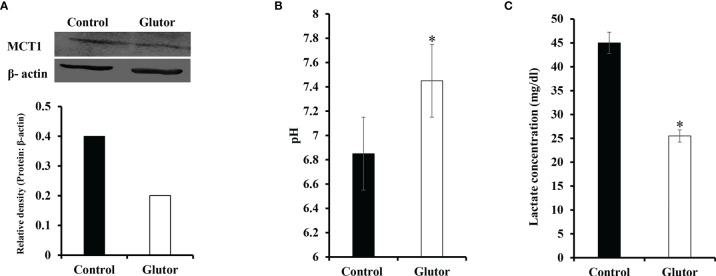
Glutor interferes with pH homeostasis of DL cells. DL cells (1 × 10^5^) were incubated for 24 h in a medium alone or containing glutor (0.01 µM), followed by an examination of MCT1 expression by Western blotting as described in the *Materials and Methods* section. The culture media of control and glutor-treated DL cells were also examined for pH and lactate levels. The plates shown in **(A)** are from a representative experiment out of at least two experiments with similar results. The accompanying bar diagram depicts the densitometry of the bands. Values in **(B)** and **(C)** are mean ± SD. *p < 0.05 vs. respective control.

Considering that glucose metabolism influences the pH homeostasis of tumor cells, we investigated the impact of glutor treatment on the expression of pH regulator monocarboxylate transporter 1 (MCT1) along with the estimation of pH and lactate of the culture medium. Glutor (0.01 µM) treatment of DL cells inhibited the expression of MCT1 compared to untreated control (**Figure 7A**). The pH (**Figure 7B**) of the culture supernatant of glutor-treated DL cells was found to be remarkably increased whereas the level of lactate was significantly decreased (**Figure 7C**) compared to the respective untreated control.

### Glutor Alters Chemosensitivity of DL Cells

**Figure 8:**
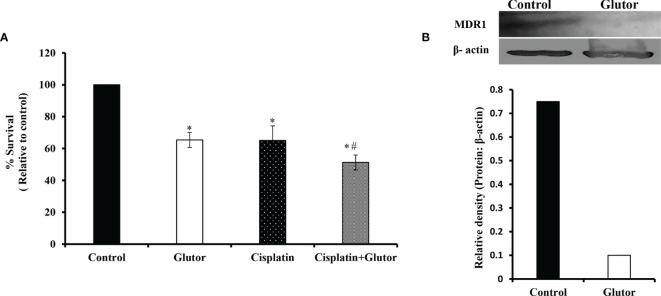
Chemosensitizing action of glutor DL cells (1 × 10^5^) were incubated in a medium alone or containing glutor (0.01 µM) for 24 h in the presence or absence of cisplatin, followed by estimation of cell survival **(A)** by MTT assay as described in the *Materials and Methods* section. Control and glutor-treated DL cells were also evaluated for MDR1 expression **(B)** by Western blotting. Values in **(A)** are the mean ± SD. The plates shown in **(B)** are from a representative experiment out of at least two experiments with similar results. The accompanying bar diagram depicts the densitometry of the bands. *p< 0.05 vs. respective control; p< 0.05 vs. DL cells treated with glutor or cisplatin alone.

As reprogrammed glucose metabolism of tumor cells is implicated in the modulation of the chemosensitivity of tumor cells, next, we examined the effect of glutor treatment on the susceptibility of DL cells to the anticancer drug cisplatin. DL cells (1 × 10^5^) were incubated in a medium alone or containing glutor (0.01 µM) in the absence or presence of cisplatin for 24 h, followed by an examination of cell survival by MTT assay and expression of multidrug resistance protein 1 (MDR1) by Western blotting as described in the *Materials and Methods* section. The treatment of DL cells with glutor plus cisplatin resulted in a significantly higher inhibition of cell survival than the DL cells incubated in a medium alone or containing cisplatin or glutor (**Figure 8A**). The expression of MDR1 decreased in DL cells treated with glutor compared to the untreated control (**Figure 8B**).

## Discussion

The present investigation demonstrates the antineoplastic and chemosensitizing action of glutor against DL cells. To understand the underlying mechanisms, we considered various possibilities. The likelihood of impaired glucose uptake due to the inhibitory action of glutor was supported by the observation of diminished glucose consumption. This observation is validated further as glutor inhibits glucose uptake in neoplastic cells of other etiologies (29, 47). Although the mechanism(s) of the inhibition of GLUT by glutor is not precisely understood, observations from our *in silico* experiments indicate a direct binding of glutor to GLUTs, possibly causing transformational alterations, which may impair glucose transport (communicated). As we observed that glutor could efficiently inhibit DL cell survival and induce apoptosis at a low IC_50_ value of 10 nM, it strongly indicates that glutor may have a high-affinity binding to GLUTs.

The pioneering work of Reckzeh et al. (29) reported the upregulated expression of GLUT1 and GLUT3 in the colorectal cancer cell line DLD-1 cultured under hypoglycemic (1 mM), no glucose condition or following treatment with glutor (0.5 µM) in the presence of glucose (25 mM). The difference with our observations could be attributed to differences in the etiology and physiology of the target cells, glucose, and glutor concentrations used. Interestingly, cell-to-cell variations has been observed in many cancer cells for GLUT expression upon the treatment with GLUT inhibitors (22, 29, 48–51).

Interestingly, we also observed downregulation of HIF-1α in glutor-treated DL cells. Moreover, tumor cells display upregulation of GLUT1 and GLUT3 expression in response to HIF-1α (52). It is noteworthy that GLUT expression is downstream of and regulated by HIF-1α (53–55). Moreover, high glucose concentrations have been reported to upregulate HIF-1α expression (56). As a result, the low glucose uptake caused by glutor-dependent-inhibition of GLUT would have triggered the observed downregulation in HIF-1α expression. Nevertheless, transcription of GLUT genes is also under the regulation of several HIF-1α independent factors, including the C-myc, K-Ras, PI3K/Akt/mTOR, cytokines, hormones, HOX transcript antisense RNA (HOTAIR), and miRNA (54, 57–64). Indeed, we also observed glutor-dependent inflection of C-myc. However, more studies will be required to interpret mechanisms underlying glutor-dependent inhibition of GLUT expression in hypoglycemic conditions.

The present observations also showed that glutor treatment of DL cells induced cell death, accompanying alterations in regulatory molecules and metabolic machinery. Indeed, our observations demonstrate mitochondrial membrane depolarization and increased ROS expression in glutor-treated DL cells. Moreover, hypoglycemia caused by impaired glucose uptake could be another trigger for induction of apoptosis. Hypoglycemia has been demonstrated to cause a decrease in ATP production associated with the induction of apoptosis (65, 66). Our experimental findings do not indicate a direct implication of glutor in the modulation of mitochondrial functions such as mitochondrial membrane potential; however, our results suggest the role of glutor-modulated p53 expression in the implication of altered mitochondrial membrane polarization. Indeed, p53 is reported to maintain mitochondrial membrane integrity and oxidative phosphorylation (67). Moreover, elevated intracellular ROS is also observed in disrupting the mitochondrial membrane potential through the AMPK/p38 MAPK signaling pathway (G. T. 68). ROS also play a vital role in activating p53 (69). Besides ROS and p53, the altered levels of Bcl-2 and BAX have also been associated with the depolarization of the mitochondrial membrane and, consequently, mitochondrial-dependent apoptosis (70–72). Moreover, p53 upregulation is implicated in glycolytic inhibition and oxidative stress-induced apoptosis in DL cells (73). Together, the experimental evidence of the present study demonstrates that glutor alters mitochondrial membrane potential in DL cells, possibly *via* increasing the levels of p53 and ROS and altering Bcl-2 and BAX levels. Nevertheless, upregulation of BAX, along with inhibition of Bcl-2, is implicated in the induction of apoptosis in neoplastic cells (74–76). Moreover, the inhibited expression of HK-2 and LDH-A in glutor-treated DL cells could also impede glycolysis. Thus, the inhibited glycolysis-associated multiple alterations could be one of the likely reasons for the induction of apoptosis.

Interestingly, intracellular ROS has been shown to inhibit various enzymes of glycolysis such as GAPDH, PDK, and PFK, strongly suggesting its crucial role in the regulation of glycolysis (77, 78). Other studies have also reported that inhibition of GLUT1 leads to increased expression of ROS (79, 80). Moreover, intracellular ROS has been demonstrated to trigger apoptosis in neoplastic cells (81).

Nevertheless, glutor treatment also modulated the expression of Hsp70 and TGF-β, which are downstream of HIF-1α and play a crucial role in regulating cell survival (82, 83). As IL-2 plays an indispensable role in T-cell proliferation (84–88), we also checked if IL2 receptor expression is modulated in DL cells following the decrease of glucose assimilation. Interestingly, glutor treatment of DL cells caused a decrease in CD25 expression, which could be possibly another reason for the observed decrease in the proliferation of DL cells. Indeed, the expression of HIF-1α impacts CD25 expression (84).

Another exciting aspect of the present study was the deregulation of pH homeostasis in glutor-treated DL cells, associated with decreased lactate production and MCT1 expression. Additionally, lactate has been shown to exert a predisposing effect on HIF-1α expression in neoplastic cells displaying the Warburg phenomenon (89). Nevertheless, p53 can also regulate MCT1 expression and hence pH homeostasis of cancer cells (90; Monde 91, 92). Moreover, modulated ROS level has been demonstrated to alter the expression of MCT1 (78, 93, 94). Even glucose levels can modulate MCT1 expression (95). Moreover, HIF-1α also regulates the expression of both MCT1 and LDH-A in cancer cells (96, 97). As we observed a decrease in HIF-1α expression, it could be one of the crucial reasons for the observed decrease in the expression of MCT1 and LDH-A in glutor-treated DL cells.

Next, we examined if glutor can alter the vulnerability of cancer cells to the chemotherapeutic agent cisplatin, considering the role of glucose metabolism in regulating chemosensitivity and MDR expression in neoplastic cells (98). Treatment of DL cells with glutor resulted in augmented cytotoxicity of cisplatin, indicating that it enhances chemosensitivity. Thus, this observation reflects the potential of a new therapeutic modality of using glutor as an adjuvant to increase the chemosensitivity of DL cells. Our observations align with earlier studies showing that p-glycoprotein expression is modulated by glucose levels rendering neoplastic cells susceptible to anticancer drugs (99, 100). Indeed, accelerated glycolysis of cancer cells has been reported to elevate ABC transporter activity in neoplastic cells (101, 102). Moreover, GLUT inhibition has been associated with altered drug efflux in cancer cells. Nevertheless, the expression of MDR molecules is also downstream of HIF-1α, which was inhibited in glutor-treated DL cells. Moreover, modulated levels of lactate have also been implicated in altering MDR1 expression (103).

**Figure 9:**
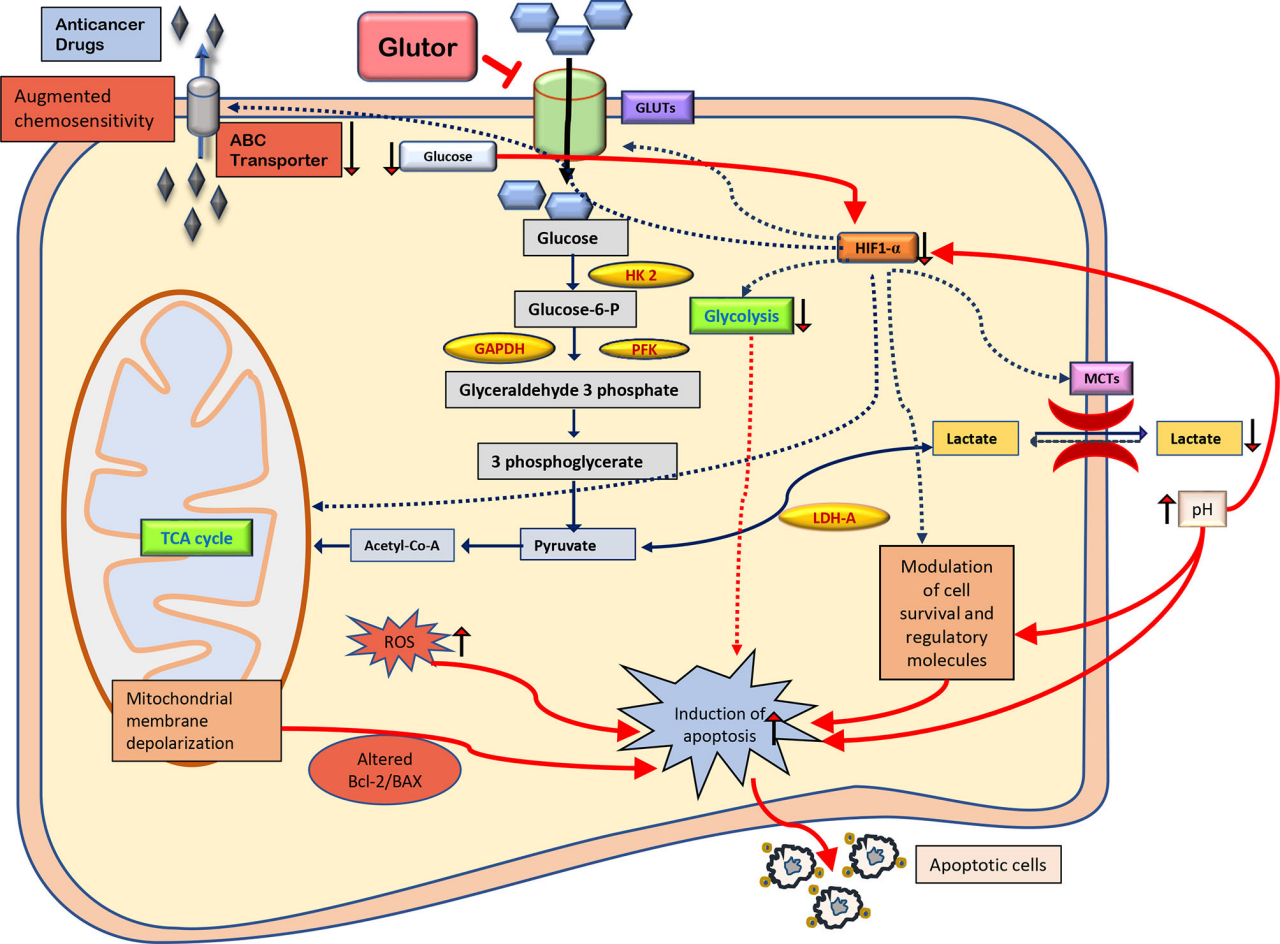
Summary of the molecular mechanisms of the antineoplastic and chemosensitizing action of glutor. The antineoplastic action of glutor involves diminished uptake of glucose, modulated expression of metabolic and cell survival regulatory molecules, altered pH homeostasis, intracellular ROS production, and chemosensitivity. Abbreviations: GLUT, glucose transporter; HK 2, hexokinase 2; GAPDH, glyceraldehyde 3-phosphate dehydrogenase; PFK, phosphofructokinase; HIF-1α, hypoxia-inducible factor 1-alpha; LDH-A, lactate dehydrogenase A; MCTs, monocarboxylate transporters; Bcl-2, B-cell lymphoma 2; BAX, Bcl-2 Associated X; ROS, reactive oxygen species; ABC transporter, ATP-binding cassette transporters.

The present study’s findings suggest that glutor exposure of thymic tumor cells can manifest hypoglycemic conditions leading to a catastrophic effect on the carbohydrate metabolism of neoplastic cells with multiple consequences. Moreover, the cytotoxic and chemosensitizing action of glutor could be facilitated by interdependent molecular events, implicating modulated expression of cell survival and metabolism regulatory molecules, pH regulation, intracellular ROS production, and MDR1 expression. It must also be noted that adding extra fructose but not glucose to the culture medium could partially reverse the inhibitory action of glutor on tumor cell survival. This observation indicates the extraordinary ability of neoplastic cells to utilize alternative fuels to compensate and revive their metabolism even if one pathway gets blocked by an inhibitor. Therefore, these observations must be considered while designing therapeutic regimens using glutor. A summary of the possible molecular mechanisms underlying glutor-dependent inhibition of tumor cell survival is depicted in **Figure 9**. This study also opens future possibilities to explore the translational value of these observations by testing the tumor growth-retarding action of glutor under *in vivo* preclinical models. Thus, these findings will have a long-lasting clinical significance in evaluating and optimizing the antineoplastic potential of glutor.

## Data Availability Statement

The raw data supporting the conclusions of this article will be made available by the authors, without undue reservation.

## Ethics Statement

The animal study was reviewed and approved by the Institutional Animal Ethical Committee, Institute of Science, Banaras Hindu University, Varanasi 221005, UP, India (Approval No. BHU/DoZ/IAEC/2021-2022/016).

## Author Contributions

MT conceived the idea, performed experiments, contributed to data generation, interpreted data, and wrote the manuscript. SY interpreted data and wrote the manuscript. YG interpreted data and wrote the manuscript. SP interpreted data and wrote the manuscript. AK interpreted data and wrote the manuscript. SS conceived the idea, interpreted data, and wrote the manuscript. All authors contributed to the article and approved the submitted version.

## Conflict of Interest

The authors declare that the research was conducted in the absence of any commercial or financial relationships that could be construed as a potential conflict of interest.

## Publisher’s Note

All claims expressed in this article are solely those of the authors and do not necessarily represent those of their affiliated organizations, or those of the publisher, the editors and the reviewers. Any product that may be evaluated in this article, or claim that may be made by its manufacturer, is not guaranteed or endorsed by the publisher.

## Supplementary Material

Supplementary Figure 1 Molecular structure of glutor Glutor is a piperazine-2-one derivative with IUPAC Name- (S)-6-Methyl-5-(4-morpholinobenzyl)-4-oxo-2-phenyl-N-(pyridin-3-ylmethyl)-4,5,6,7-tetrahydropyrazolo[1,5-a]pyrazine-6-carboxamide.
