# Supplementary figures and images for: Glutor, a Glucose Transporter Inhibitor, Exerts Antineoplastic Action on Tumor Cells of Thymic Origin: Implication of Modulated Metabolism, Survival, Oxidative Stress, Mitochondrial Membrane Potential, pH Homeostasis, and Chemosensitivity
Source: Front Oncol. 2022 Jun 30;12:925666. doi: 10.3389/fonc.2022.925666 (PMC9279700; doi:10.3389/fonc.2022.925666)

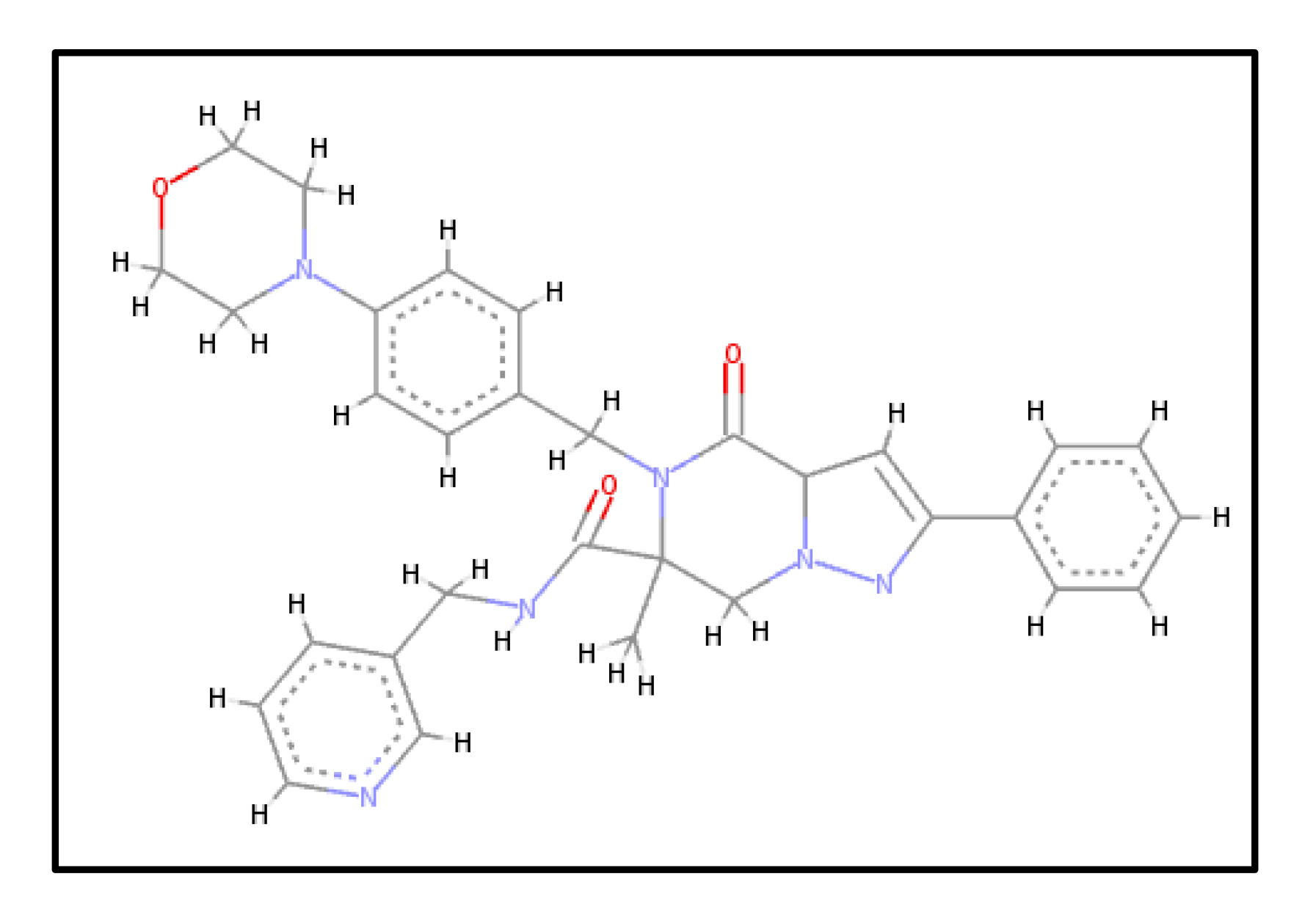

Supplement: Supplementary Figure 1 — Molecular structure of glutor Glutor is a piperazine-2-one derivative with IUPAC Name- (S)-6-Methyl-5-(4-morpholinobenzyl)-4-oxo-2-phenyl-N-(pyridin-3-ylmethyl)-4,5,6,7-tetrahydropyrazolo[1,5-a]pyrazine-6-carboxamide. [file Image_1.tif]
